# Higher Adherence to the Mediterranean Diet Is Associated With Preserved White Matter Integrity and Altered Structural Connectivity

**DOI:** 10.3389/fnins.2020.00786

**Published:** 2020-08-12

**Authors:** Belina Rodrigues, Ana Coelho, Carlos Portugal-Nunes, Ricardo Magalhães, Pedro Silva Moreira, Teresa Costa Castanho, Liliana Amorim, Paulo Marques, José Miguel Soares, Nuno Sousa, Nadine Correia Santos

**Affiliations:** ^1^Life and Health Sciences Research Institute (ICVS), School of Medicine, University of Minho, Braga, Portugal; ^2^ICVS/3B’s – PT Government Associate Laboratory, Braga, Portugal; ^3^Clinical Academic Center – Braga, Braga, Portugal

**Keywords:** Mediterranean diet adherence, structural connectivity, white matter integrity, Mediterranean Diet Assessment Screener, healthy aging

## Abstract

The Mediterranean diet (MedDiet) has been associated with cognitive performance. Yet, controlled trials have yielded contradictory results. To tackle this controversy, a comprehensive multimodal analysis of the association of the MedDiet with cognitive performance and brain structure in normative aging is still necessary. Here, community dwellers ≥50 years from a cohort study on normative aging (*n* = 76) underwent a (i) magnetic resonance imaging session with two acquisitions: structural and diffusion-weighted imaging (DWI); (ii) neuropsychological battery of tests focusing on memory and executive functioning; and (iii) dietary assessment through the Mediterranean Diet Assessment Screener (MEDAS, score range: 0–14, scores ≥10 indicate high adherence to the Mediterranean diet) 18 months prior to the brain imaging and neuropsychological assessment. We found that high adherence to the MedDiet (MEDAS ≥10) was associated with higher values of fractional anisotropy and lower diffusivity values in the brain white matter. Similarly, high adherence to the MedDiet was associated with higher structural connectivity between left hemisphere brain regions. Specifically, the amygdala, lingual, olfactory, middle occipital gyrus, and calcarine areas. No association was found between high adherence to the MedDiet and total brain volumes or hypointensities. Higher adherence to the MedDiet was positively associated with executive functioning scores. These results suggest that high adherence to the MedDiet positively associates with brain health, specifically with executive function scores and white matter integrity of bundles related to the processing and integration of taste, reward, and decision making. These findings seem to support the view that the MedDiet should be part of recommendations to promote a healthy brain.

## Introduction

The Mediterranean diet (MedDiet) is a dietary pattern characterized by high consumption of vegetables, fruits, legumes, and cereals, use of olive oil as the main culinary fat, moderate consumption of alcohol, and low consumption of red meat and dairy products (Trichopoulou et al., 2014). Observational studies show a positive association of the MedDiet with cognitive scores including general cognitive function, memory, and executive function (Wengreen et al., 2013; Qin et al., 2015; Hardman et al., 2016). However, randomized controlled trials reported no significant differences between the MedDiet group and the comparison group (Knight et al., 2016; Marseglia et al., 2018). A multimodal neuroimaging approach might provide clarification as to apparent unsuccessful translation from observational studies to clinical trials.

Studies in older adults applying a multimodal neuroimaging approach and neuropsychological testing have shown that the MedDiet is associated with several brain correlates, such as global volumes and white matter integrity, but not always with cognitive performance. For instance, higher adherence to the MedDiet was associated with lower diffusivity values as measured by diffusion tension imaging, which, in turn, was linked to memory scores (Pelletier et al., 2015). Yet, no association was detected in other brain correlates reported to be related with cognitive decline such as the total volume of gray and white matter, or hyperintensities (Pelletier et al., 2015). In contrast, high adherence to the MedDiet was associated with larger gray matter volumes (GMVs) of the dentate gyrus, but not with hyperintensities (Karstens et al., 2019). Interestingly, in this case, high adherence to the MedDiet was also associated with higher learning and memory composite score, but the GMV of the dentate gyrus did not mediate the association between the MedDiet and cognition (Karstens et al., 2019). These findings suggest that the MedDiet potentially exerts its effects through diverse mechanisms that can only be fully understood by resorting to a multimodal neuroimaging approach.

Even though several studies of the association of the MedDiet with cognition have been done regarding brain volumes and hyperintensities volumes, few studies have also focused on the white matter integrity (Pelletier et al., 2015) and none on the brain structural connectivity. An approach combining dietary assessment, neuropsychological evaluation, and several neuroimaging brain-based measures has the potential to provide complementary information in understanding the relationship between eating patterns, brain structure, and cognition. Here, the present work aims to address whether MRI-based measures differ among aging community-dweller older adults as a function of the MedDiet adherence. We hypothesized that high adherence to the MedDiet was associated with larger global brain volumes, preserved white matter integrity, and higher structural connectivity.

## Materials and Methods

### Participants

**FIGURE 1:**
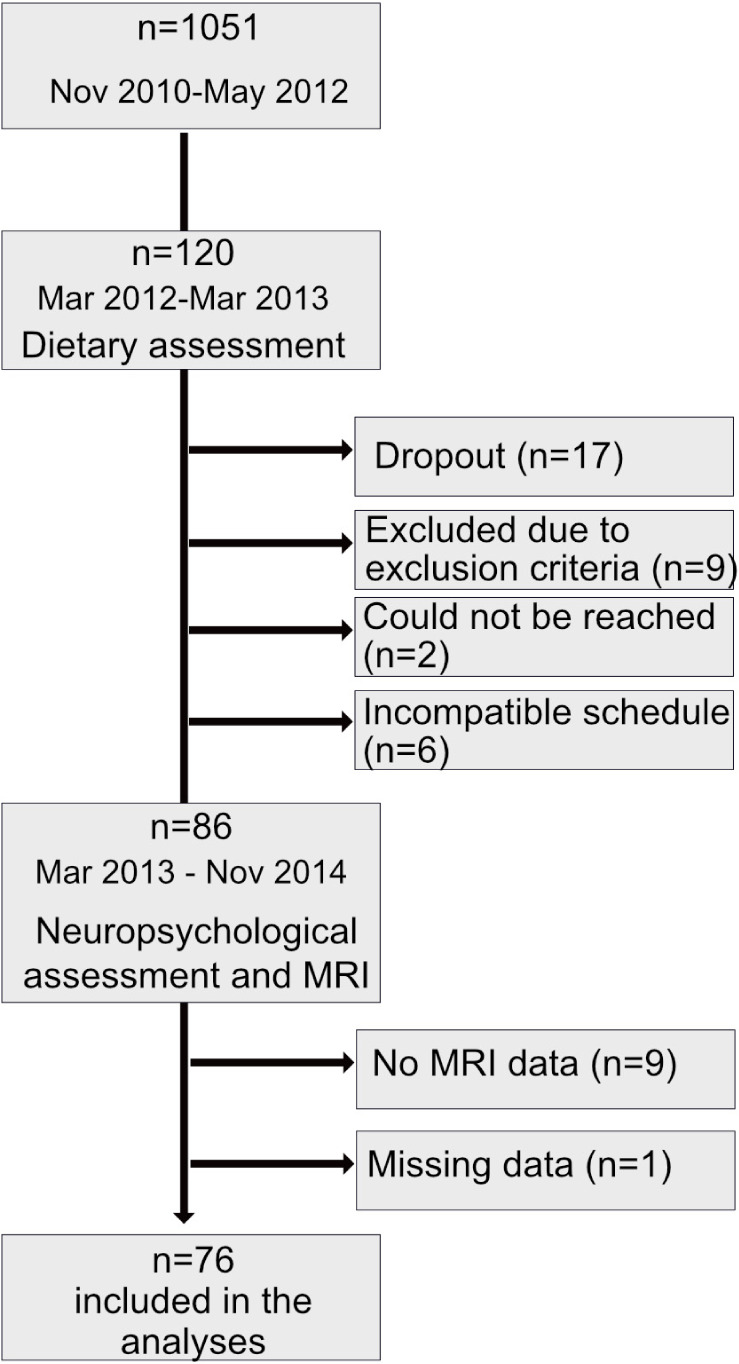
Sample size and timeline of procedures.

Participants are part of the sample recruited for the SWITCHBOX Consortium project.^1^ Briefly, community-dwelling participants aged 50 or older (*n* = 1,051, final sample size after exclusion criteria) were recruited from the local area health authority registries. The primary exclusion criteria included inability to understand informed consent, participant choice to withdraw from the study, incapacity and/or inability to attend the clinical and neuropsychological assessment session(s), dementia, or diagnosed neuropsychiatric or neurodegenerative disorder (medical records). A team of clinicians performed a standardized clinical interview that included current medication and allowed to further detect and exclude disorders of the central nervous system (epilepsy and neurodegenerative disorders) as well as overt thyroid pathology, and a team of psychologists carried out the neuropsychological assessment (Santos et al., 2014). Of the original 1,051 participants, 246 individuals were randomly selected and contacted. Of these, 120 integrated the prospective cohort whose main aim was to study the healthy cognitive aging. From these, 86 individuals accepted to be reevaluated for follow-up. Of these, 77 performed the MRI acquisition protocol, but 1 was excluded due to missing data, which resulted in a total of 76 individuals (Figure 1). Clinical assessments took place at the Clinical Academic Center – Braga (2CA-B; Braga, Portugal). All participants provided informed consent to participate in the present study. The study was approved by the national and local ethics review boards and conducted in accordance with the Declaration of Helsinki.

### Dietary Assessment

The dietary assessment was measured on an average of 18 months (range 16–25 months, 10.2% > 19 months) prior to the MRI scan, and neuropsychological assessment and consistency of the dietary pattern was confirmed by a non-significant paired *t*-test between the two moments. To ascertain the total energy intake, a 24-h dietary recall was applied, whereas the adherence to the MedDiet was measured through the Mediterranean Diet Adherence Screening (MEDAS). As for the 24-h recall, a manual adaptation of the Automated Multiple-Pass Method (Raper et al., 2004) was applied by an experienced dietician. After obtaining a list of all food items eaten on the previous day, the interviewer probed for frequently forgotten foods such as savory snacks or candies. Then, the time and name of the eating occasion (e.g., breakfast) and a detailed description of the type of food item, cooking method, and other relevant aspects were collected. At last, a final revision of the dietary intake took place (Raper et al., 2004). Once the data were collected, energy intake was determined by Nutrilog SAS software (version 3.1). For this analysis, a single 24-h recall was collected.

Mediterranean Diet Adherence Screening is a 14-item questionnaire (12 questions on food consumption frequency and 2 on food intake habits) aiming at measuring adherence to the MedDiet (Schröder et al., 2011). Each question is scored according to the cutoff points of each item that better represented the observed dose–response relationships between the MedDiet and a statistically significant protection against myocardial infarction in a case-control study (Martinez-Gonzalez et al., 2002). Thus, one point is assigned when one (1) uses olive oil as the principal source of fat for cooking, (2) prefers white over red meat, and (3) eats a dish with sauce of tomato, garlic, onion, or leeks sautéed in olive oil twice per week. One point is also assigned when one consumes: (1) ≥4 tablespoons of olive oil/day; (2) ≥2 servings of vegetables/day; (3) ≥3 pieces of fruit/day; (4) <1 serving of red meat, hamburger, or sausages per day; (5) <1 serving of animal fat/day; (6) <1 cup of sugar-sweetened beverages/day; (7) ≥7 servings of wine/week; (8) ≥3 servings of pulses/week; (9) ≥3 servings of fish or seafood per week; (10) <2 commercial pastries/week; and (11) ≥3 servings of nuts/week. In case the conditions are not met, 0 points are recorded. The final score ranges between 0 and 14 points, and values equal to, or greater than, 10 ensure high adherence (Martínez-González et al., 2012). Whereas item 1 (use of olive oil as the principal source of fat for cooking) and item 2 (preference for white over red meat) measure food intake habits, all other items measure food consumption frequency. Thus, adherence to the MedDiet was dichotomized as per the cutoff of 10, yielding two groups: high adherence to the MedDiet (MEDAS ≥ 10) and low adherence to the MedDiet (MEDAS < 10) (Martínez-González et al., 2012). MEDAS reliably ranks the MedDiet adherence (Schröder et al., 2011; Papadaki et al., 2018) with a moderate correlation with dietary intake (Schröder et al., 2011), corroborated with objective blood biomarkers (Hebestreit et al., 2017).

### Neuropsychological Assessment and Cognitive Scores

Trained psychologists carried out the neuropsychological evaluation. The battery of tests was selected to assess specific cognitive profiles (general cognition, executive and memory functions). The mini-mental state examination (MMSE) evaluated the global cognitive status (Folstein et al., 1975), the multiple trial verbal learning – the selective reminding test (SRT) assessed memory function (Buschke et al., 1995); and Stroop color and word test (STROOP) measured executive function with response inhibition/cognitive flexibility (Strauss et al., 2006). Then, two latent variables were defined: executive function and memory. These two latent variables were previously validated through a confirmatory factor analysis, which found that a two-factor solution composed by a so-termed memory factor (MEM) and general and executive factor (GENEXEC) better represented the factor structure of the neuropsychological measures (Santos et al., 2015). Executive functioning included the MMSE and STROOP, whereas memory performance resorted to SRT (Moreira et al., 2018). The final scores of each subtest, i.e., STROOP (words, colors, words/colors) and SRT (memory score long-term storage, consistent-term retrieval, and delayed recall), were transformed according to the proportion of maximum scaling, where each test score was subtracted from the minimum of the sample and divided by the difference between the maximum and the minimum (Moreira et al., 2018).

### Brain Measures

#### MRI Data Acquisition

The brain imaging sessions took place at the Hospital of Braga (Portugal) on a clinically approved Siemens Magnetom Avanto 1.5 T MRI scanner (Siemens Medical Solutions, Erlangen, Germany), using a 12-channel receive-only head coil. Two acquisitions comprising the imaging protocol were considered in this study: structural and diffusion-weighted imaging (DWI). For the structural acquisition, a 3D T1-weighted magnetization prepared rapid gradient echo (MPRAGE) sequence was used with the following parameters: 176 sagittal slices, TR/TE = 2,730/3.48 ms, flip angle = 7°, slice thickness = 1.0 mm, in-plane resolution = 1.0 × 1.0 mm^2^, FoV = 256 × 256 mm, slice gap = 0 mm. As for the DWI scan, a spin-echo echo-planar imaging (SE-EPI) sequence was used with the following parameters: TR = 8,800 ms, TE = 99 ms, FoV = 240 × 240 mm, acquisition matrix = 120 × 120, 61 2-mm axial slices with no gap, 30 non-collinear gradient directions with b = 1,000 s/mm^2^, one b = 0 s/mm^2^ acquisition, and two as total number of repetitions.

Prior to data preprocessing, all acquisitions were visually inspected by the authors, including a certified neuroradiologist, to confirm that none of the participants had brain lesions nor critical head motion or artifacts that could compromise data quality.

#### MRI Data Pre-processing

##### WM and GM volumes

Structural scans were processed using the Statistical Parametric Mapping – SPM12 – (Welcome Department of Imaging Neuroscience, Institute of Neurology, London, United Kingdom) using MATLAB version R2016a (The MathWorks Inc., United States). First, DICOM files were converted to NIfTI format, and each participant’s anatomical image was centered and then segmented into gray, white matter and cerebrospinal fluid using the SPM12 segmentation tool. Individual images were then coregistered between participants using Diffeomorphic Anatomical Registration Through Exponentiated Lie Algebra (DARTEL). Following the alignment of the gray and white matter, the registered images were normalized to the Montreal Neurological Institute (MNI) stereotactic space using the DARTEL template and spatially smoothed using a Gaussian kernel with full width at half maximum of 8 mm. Total values of gray matter volume (TGMV), white matter (TWMV), and cerebrospinal fluid (CSF) were obtained from the sum of the product of the voxel value and the voxel volume. Total intracranial volume (ICV) was obtained from the sum of the GM, WM, and CSF volumes.

##### White matter hypointensities volume

White matter hypointensities (WMH) were estimated after white matter segmentation and white matter signal abnormalities (WMSA) volume estimation of structural images. Briefly, the standard semi-automated workflow implemented in Freesurfer toolkit version 5.1^2^ comprises 31 processing steps, which include the spatial normalization to Talairach standard space, skull stripping, intensity normalization, tessellation of GM–WM boundary, and cortical, subcortical, and WM segmentation. Freesurfer identifies the WMSA using probabilistic procedures that were extended to WM lesion identification (Fischl et al., 2002). This pipeline is validated against manual segmentations (Fischl et al., 2002) and correlates with estimates based on FLAIR acquisitions (Bagnato et al., 2010).

##### White matter integrity/structural connectivity

WM microstructure was studied through diffusion tensor imaging (DTI) using tract-based spatial statistics (TBSS) pipeline^3^ (Behrens et al., 2003), within the FMRIB Software Library (FSL v5.0^4^). All DWI preprocessing was performed using FMRIB’s Diffusion Toolbox (FDT) also provided with FSL and included the following steps: (i) eddy current distortions and movement correction, (ii) matching rotation of the diffusion directions, and (iii) isolation of brain signal by extraction of the skull.

Following the preprocessing, the diffusion tensor was fitted to the data, and the scalar maps were computed using DTIFIT. DTIFIT is part of the FDT Toolbox and fits a diffusion tensor model at each voxel and generates the scalar maps of fractional anisotropy (FA) and mean diffusivity (MD), and eigenvector and eigenvalue maps. Axial diffusivity (AD) scalar map was defined as the principal diffusion eigenvalue (L1) and radial diffusivity (RD) as the mean of the second and third eigenvalues [(L2 + L3)/2].

To generate the structural connectivity matrices, probabilistic tractography was used. The following steps were applied: (i) local modeling of the diffusion parameters was conducted using bedpostx, which runs Markov Chain Monte Carlo sampling; (ii) normalization of the automated anatomical labeling (AAL) atlas regions of interest (ROIs) to each participant native diffusion space; and (iii) probabilistic tractography was run using probtrackx2, which is part of the FDT toolbox; AAL ROIs in participants’ diffusion space were used as seed masks, and 5,000 streamlines were sampled from each voxel in the seed mask.

### Covariates

Age, sex, number of years of formal education, and data on lifestyle factors such as physical activity and smoking habits were also collected. The physical activity level was assessed through the International Physical Activity Questionnaire—Short form (IPAQ-SF) that assesses the types of the intensity of physical activity and sitting time over the previous 7 days. Estimates are given in MET-min/week and time spent sitting (Craig et al., 2003). As for the smoking habits, participants were categorized as non-smoker, former smoker, or current smoker. In addition, the mood and the body mass index (BMI) were also assessed. Depressive mood status was ascertained by the Geriatric Depression Scale (GDS) (Yesavage et al., 1982). The GDS is a 30-item questionnaire, whose scores range from 0 to 30 representing the total of depressive symptoms. The BMI was calculated according to the standardized manual based on international recommendations (Stewart et al., 2011) by measuring weight (Tanita BF 350 Body Composition Analyzer; Tanita Corporation, Tokyo, Japan) and height (stadiometer Seca 217; Seca GmBH, Hamburg, Germany), and computing as weight in kilograms divided by the square of height in meters.

### Data Analysis

Diffusion structural connectivity analyses resorted to a connectomics approach, and the networks were built using the AAL atlas. To estimate the structural connectivity based on the diffusion data, the probtrackx tool from the FDT toolbox (Behrens et al., 2007) was used. Thus, the structural connectivity was estimated by computing the number of streamlines, i.e., white matter fibers connecting gray matter regions, connecting each pair of regions of interest from the atlas, through sampling the principle directions previously calculated at each voxel. Per voxel, 5,000 streamlines were attempted, which resulted in a matrix of streamlines connecting each pair of ROIs. In turn, this matrix was normalized by (i) dividing each streamline by the way total value of the respective seed; this value corresponds to the total number of generated tracts from the seed mask that have reached at least one of the other masks and have not been rejected by inclusion/exclusion mask criteria; and (ii) averaging the upper and the lower triangles to obtain an undirected connectivity matrix. Then, the matrix was filtered by applying a one-sample *t*-test to each individual connection, and only connections with *p* < 0.05 corrected for multiple comparisons with false discovery rate (FDR) were kept.

Voxel-wise statistical analysis of scalar maps was performed using TBSS procedures (Smith et al., 2006), implemented in FSL. First potential outliers from diffusion tensor fitting were removed by eroding the FA maps of each participant and zeroing the end slices. Then, a non-linear registration procedure was applied to align all FA images to a 1 × 1 × 1-mm standard space. To find the most representative FA image (i.e., the one requiring the least warping to align all images) that served as the study-specific template, the FA image from each participant was non-linearly registered to each other. Then, after this template image was affine-transformed into MNI 152 standard space, each FA map was transformed into standard space by combining the non-linear transformation to the FA target with the affine transformation into MNI space. Then, the skeleton image was obtained by skeletonizing the average of all FA images. The resultant skeleton image was thresholded at 0.3 so that skeleton regions, including multiple tissue types, could be removed. Finally, all scalar maps (FA, MD, AD, and RD) were projected onto the mean FA skeleton using the transformations applied to the FA images.

### Statistical Analyses

Descriptive statistics were computed and reported as mean ± standard deviation for the continuous variables and number and percentages for categorical variables. Statistical differences between groups were studied through the Welch’s test due to the unequal groups’ sizes for continuous variables and x^2^ test for categorical variables.

To examine the association between the adherence to the MedDiet and cognitive scores, a multiple linear regression was performed. After the study of the statistical assumptions, the association of the adherence to the MedDiet, as measured by MEDAS (continuous variable) with memory and executive scores, was studied while controlling for age, sex, years of education, GDS score, BMI, occupation, and energy intake.

To study the association between adherence to the MedDiet and total brain volumes and WMH, multiple linear regressions were performed. The three regression models tested accounted for (1) time between dietary assessment and MRI scans (TBA) and intracranial volume (ICV); (2) TBA, ICV, and age; and (3) TBA, ICV, age, sex, years of education, and BMI. These analyses were rerun, excluding participants with implausible intake, i.e., participants whose energy intake is less than 500 kcal/day and greater than 3,500 kcal/day (Rhee et al., 2015). In these analyses, two participants were excluded based on implausible intake criteria (Supplementary Material). The descriptive analyses, independent *t*-tests, x^2^, and linear regressions were computed using SPSS version 24 (IBM, SPSS, Chicago, IL, United States).

To assess structural integrity and its relationship with the adherence to the MedDiet, a model, including group assignment (i.e., Low MEDAS vs. High MEDAS), time between dietary assessment and MRI scans, age, sex, years of education, and BMI, was built, and the network-based statistic (NBS) procedure implemented in the NBS toolbox^5^ was applied. The NBS tests hypotheses about the human connectome by evaluating the null hypothesis at the level of interconnected edges (i.e., subnetworks) surviving a predefined primary threshold as opposed to considering the null hypothesis at the single edge level. First, it tests the hypotheses at each individual connection in the network and thresholds it with a user-defined threshold (primary threshold). Then, subnetworks composed of connections whose significance exceeds the primary threshold are identified, and its significance is determined. The subnetwork significance is calculated by comparing their sizes to the distribution of the size of subnetworks obtained through 5,000 random permutations of the original hypothesis. To capture different effects, it is recommended to use different primary thresholds (Zalesky et al., 2010). Thus, in the present study, five different primary thresholds were used (*p* < 0.01, p < 0.005, *p* < 0.001, *p* < 0.0005, *p* < 0.0001) to capture less pronounced, but more extent, effects (less stringent primary threshold – *p* < 0.01) as well as localized and pronounced effects (most stringent threshold – *p* < 0.0001). Five thousand permutations were performed, and networks were considered significant at a network size corrected level of *p* < 0.05. BrainNet Viewer^6^ was used for visualization of the significant networks.

The statistical analysis of the skeletonized maps of FA, MD, AD, and RD was performed using the permutation-based cross-participant statistics methods implemented randomly (Winkler et al., 2014) and distributed with FSL. The same model was used as in the structural connectivity analysis. Ten thousand random permutations were used in the inference of the contrasts of interest. Threshold-free cluster enhancement (TFCE) was applied to detect widespread significant differences, and family-wise error (FWE) correction at *p* < 0.05 was used to correct for multiple comparisons. The projected regions showing significant results were then labeled according to the John Hopkins University ICBM-DTI-81 WM labels atlas (Hua et al., 2008) distributed with FSL. For visualization purposes, the significant tracts were dilated using the tbss_fill tool (distributed with FSL).

## Results

### Characterization of the Sample

**FIGURE 2:**
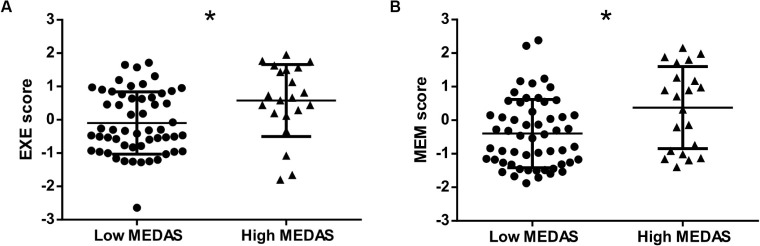
Cognitive scores by adherence to the Mediterranean diet (MedDiet). Both executive functioning scores **(A)** and memory scores **(B)** were statistically higher in those with high adherence to the MedDiet [high Mediterranean Diet Assessment Screener (MEDAS)]. EXE, executive functioning; MEDAS, Mediterranean Diet Assessment Screener; MEM, memory. Asterisks indicate that cognitive scores reached statistical significance in participants with low MEDAS vs. participants with higher MedDiet adherence (*p* < 0.05).

Participants were, on average, 66.4 (±7.75) years old, with 48.7% being women. Two-thirds were retired, and 65.8% were non-smokers. When comparing the participants with low adherence to the MedDiet, those with high adherence had a higher number of formal years of education, lower BMI (Table 1), and higher cognitive (both memory and executive function) scores (Figure 2). The two groups did not display other statistical differences.

**TABLE 1:** Demographic and cognitive profile of the full cohort and grouped by low vs. high adherence to the Mediterranean diet (MedDiet).

	All	Low MEDAS	High MEDAS	Test statistic
Sample size	76	55	21	
Age, years	66.4 (7.75)	66.9 (7.60)	65.0 (8.16)	F_welch_(1,34.1) = 0.83, *p* = 0.37, ω^2^ = -0.002
Female, n, (%)	37 (49.3)	30 (54.5)	7 (33.3)	X^2^_1_,_76_ = 2.74, *p* = 0.13, φ = 0.19
Education, years	5.83 (4.07)	5.1 (3.60)	7.6 (4.74)	F_welch_(1,29.2) = 4.69, *p* = 0.04^a^, ω^2^ = 0.04
Energy intake, kcal	2132 (824)	2040 (704)	2377 (1061)	F_welch_(1,25.6) = 1.73, *p* = 0.20, ω^2^ = 0.01
GDS, score	9.45 (6.75)	10.1 (6.93)	7.65 (6.03)	F_welch_ (1,39.1) = 2.26, *p* = 0.14, ω^2^ = 0.02
BMI, kg/m^2^	29.4 (3.66)	30.0 (3.55)	27.6 (3.38)	F_welch_(1,37.9) = 7.76, *p* = 0.01^a^, ω^2^ = 0.08
Physical activity, MET min/week	561 (584)	486 (548)	757 (644)	F_welch_(1,31.7) = 2.89, *p* = 0.10, ω^2^ = 0.02
**Smoking habits, n, (%)**				
Non-smoker	50 (65.8)	39 (70.9)	11 (52.4)	X^2^_1_,_76_ = 2.61, *p* = 0.25, *V* = 0.18
Former smoker	20 (26.3)	12 (21.8)	8 (38.1)	
Smoker	6 (7.9)	4 (7.3)	2 (9.5)	
**Occupation status, n, (%)**				
Employed	14 (18.4)	9 (16.4)	5 (23.8)	X^2^_1_,_76_ = 0.82, *p* = 0.68, *V* = 0.11
Retired	56 (73.7)	41 (74.5)	15 (71.4)	
Unemployed	6 (7.9)	5 (9.1)	1 (4.8)	
**Neuropsychological assessment**				
Memory scores	-0.185 (1.13)	-0.399 (1.02)	0.375 (1.22)	F_welch_(1,31.1) = 6.63, *p* = 0.02^a^, ω^2^ = 0.07
Executive functioning	0.090 (1.02)	-0.096 (0.935)	0.577 (1.08)	F_welch_(1,32.1) = 6.33, *p* = 0.02^a^, ω^2^ = 0.07

*Values are mean (SD) unless otherwise specified. GDS, geriatric depression scale; BMI, body mass index; MET, metabolic equivalent. ^a^p < 0.05.*

### Association of the MedDiet Scores With Cognitive Scores

In a multiple linear regression adjusted for age, sex, years of education, GDS score, BMI, occupation, and energy intake, higher adherence to the MedDiet was associated with higher executive scores (Table 2). More specifically, each unit increase in MEDAS corresponded to the same magnitude of effect as that observed for 0.5 years of school (Supplementary Table S1). Unlike executive functioning scores, a positive trend was found between higher adherence to the MedDiet and memory scores (*p* = 0.06) (Table 2).

**TABLE 2:** Association between adherence to the Mediterranean diet, as measured by the Mediterranean Diet Assessment Screener (MEDAS) as continuous variable and the cognitive scores.

				95% Confidence interval	
	B	β	*p*-Value	Lower bound	Upper bound
Executive functioning scores^a^	0.118	0.232	0.02^c^	0.020	0.215
Memory scores^b^	0.121	0.213	0.06	–0.006	0.247

*Results are presented as B (unstandardized coefficient) and β (standardized coefficient), controlled for age, sex, years of education, GDS, BMI, occupation, energy intake, and physical activity. Test statistic: ^a^-F(9,62) = 7.25, p < 0.0001, adj. R^2^ = 0.45. ^b^-F(9,62) = 3.67, p = 0.001, adj. R^2^ = 0.27. ^c^p < 0.05.*

### Association of the MedDiet Scores With Brain Volumes

In multiple linear regression adjusted for time between assessments and ICV (model 1), a trend was found between higher adherence to the MedDiet and larger TGMV (*p* = 0.051) (Table 3 and Supplementary Table S3). In model 2, a 1-year increase in age was associated with a reduction in 0.003 L of GMV (*p* < 0.001) (Table 3 and Supplementary Table S4), indicating that the average difference in GMV between high and low MedDiet (0.28 L) was of the magnitude of effect corresponding to the change in TGMV during 8 years of aging. Yet, when further adjusted for time between assessments, sex, years of education, and BMI (model 3), higher adherence to the MedDiet was no longer associated with TGMV (Table 3). As for the TWMV and HypoV, no association was found in the studied models (Table 3).

**TABLE 3:** Association between the Mediterranean diet scores, as measured by MEDAS as dichotomous variables, i.e., high MEDAS vs. low MEDAS, and global brain volumes.

	TGMV (L)		TWMV (L)		HypoV (mm^3^)	
	B	p	B	P	B	p
Model 1	0.026	0.051	−0.011	0.361	−1,578	0.120
Model 2	0.025	0.043^a^	−0.012	0.235	−1,457	0.073
Model 3	0.010	0.394	−0.019	0.090	−957	0.284

*MEDAS coded as 0 for low adherence and 1 for high adherence. Results are presented as B (unstandardized coefficient) and p-values. Test statistics for each model are shown in Supplementary Table S6. Model 1 – adjusted for ICV; Model 2 – adjusted for ICV and age; Model 3 – adjusted for ICV, age, BMI, time between assessments, years of education, and sex. ICV, total intracranial volume; TGMV, total gray matter volume; TWMV, total white matter volume; HypoV, hypointensity volumes. ^a^p < 0.05.*

### Higher MedDiet Scores Associated With White Matter Integrity

**FIGURE 3:**
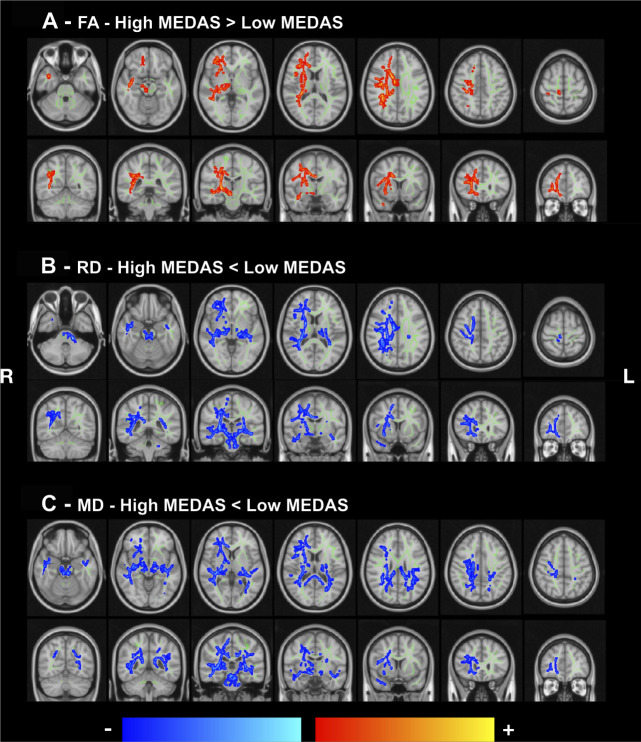
White matter integrity as a function of the MedDiet adherence. Statistically significant group differences in FA **(A)**, RD **(B)**, and MD **(C)** projected maps, controlled for body mass index (BMI), years of education, time between assessments, sex, and age. Blue/light-blue gradient indicates lower values for the high adherence group when compared to the low adherence group. Red/yellow gradient indicates higher values for the higher adherence group when compared to the low adherence group. Significance threshold was set to *p* < 0.05 (FWE corrected for multiple comparisons). **(A)** High MedDiet score is associated with higher fractional anisotropy. **(B,C)** Low MedDiet score is associated with higher radial and mean diffusivity. MEDAS, Mediterranean Diet Assessment Screener; FA, fractional anisotropy; RD, radial diffusivity; MD, mean diffusivity; R, right; L, left.

In a voxel-based TBSS analysis adjusted for time between assessments, age, sex, years of education, BMI, and controlling for multiple comparisons, a higher MedDiet score was associated with higher fractional anisotropy (FA) values in several WM areas, including the corpus callosum, right superior longitudinal fasciculus, and corona radiata (Figure 3 and Supplementary Table S7). Similarly, lower values of RD and MD were associated with high adherence to the MedDiet (Figure 3 and Supplementary Tables S8, S9). Neurofiber tracts with decreased RD and MD included cerebellar peduncle (middle, superior), the body and splenium of the corpus callosum, internal capsule (posterior limb and retrolenticular), corona radiata, cingulum, external capsule, posterior thalamic, sagittal stratum, and superior longitudinal fasciculus (Supplementary Tables S8, S9).

### Adherence to the MedDiet Is Associated With Increased Structural Connectivity

**FIGURE 4:**
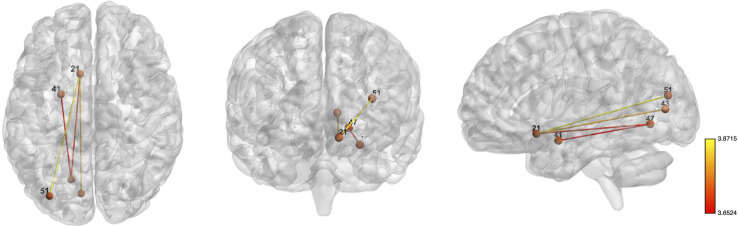
Whole-brain networks with increased structural connectivity in high adherence MedDiet group. Whole-brain networks with increased structural connectivity in high-adherence MedDiet group (when compared to the low-adherence MedDiet group), with *p* < 0.0005. High adherence to the MedDiet had higher SC in a network with the following regions: left olfactory cortex, left amygdala, left calcarine, left lingual gyrus, and left middle occipital gyrus. Color scheme: higher connectivity from red to yellow. The analyses were controlled for age, sex, years of education, and BMI. Numbers’ legend: (21) left olfactory cortex, (41) left amygdala, (43) left calcarine, (47) left lingual gyrus, and (51) left middle occipital gyrus.

As for the pattern of structural connectivity in the brain (Figure 4), participants with high adherence to the MedDiet had a higher number of white fibers connecting a network in the left hemisphere for a threshold of *p* < 0.0005; such a network, with five connections, is composed of the olfactory cortex, amygdala, calcarine, lingual, and middle occipital gyri (M_MEDAS<10_ = 0.0022 vs. M_MEDAS≥10_ = 0.0066, *p* = 0.046, *d* = 0.642). These results were controlled for time between assessments, age, years of education, sex, and BMI. No significant results were found for the threshold 0.01, 0.005, 0.001, and 0.0001.

## Discussion

Mixed findings on the association of the MedDiet with cognitive function might be clarified by combining a multimodal neuroimaging approach with neuropsychological and dietary assessments. Thus, the present work focused on MRI-based measures as a function of the MedDiet adherence. This study reveals that a higher adherence to the MedDiet is associated with higher executive functioning scores. Moreover, it shows an association between adherence to the MedDiet and the microstructure of WM, as well as higher structural connectivity in a specific brain network. Yet, the multiple regression analysis showed that high adherence to the MedDiet was not associated with global brain volumes nor HypoV. These findings suggest that the MedDiet is associated with brain correlates that have been linked with cognitive performance (Hedden et al., 2016).

Our results are in line with studies showing that higher adherence to the MedDiet is associated with cognitive scores and white matter integrity (Pelletier et al., 2015; Karstens et al., 2019). As for the cognitive scores, similar to that of McEvoy et al. (2019), high MEDAS was associated with executive functioning but not with memory scores. These findings might be related to the age of our cohort as executive functioning appears to decline earlier than other cognitive domains (McEvoy et al., 2019). Our results also confirm that high adherence to the MedDiet was associated with lower diffusivity values, including MD and RD (Pelletier et al., 2015). Interestingly, we found that differences in WM integrity, despite global, were also present in bundles linking limbic regions with the prefrontal cortex, including corpus callosum, internal capsule, cingulum, corona radiate, and superior longitudinal fasciculus. As these brain areas are associated with reward and cognitive processes, and an association was found between obesity and WM integrity of bundles within the limbic system and tracks connecting the temporal and frontal lobe (Kullmann et al., 2015), it is possible that such alterations of the WM partially are associated with cognition and eating behaviors.

The observation herein reported that the MedDiet is associated with brain structural connectivity offers an important insight into the relationship between dietary patterns and brain structure. Participants with high adherence to the MedDiet displayed higher structural connectivity in a network linking the olfactory cortex, amygdala, calcarine, lingual, and middle occipital gyri on the left hemisphere. Such anatomical pattern, together with the fact that fiber length and numbers influence synaptic transmission (Honey et al., 2009), allows us to speculate that this structural connectivity pattern might be related to the integration of sensory stimuli and reward. For instance, odor (Seubert et al., 2013), visual food cues (Huerta et al., 2014), and taste stimuli (Veldhuizen et al., 2011; Huerta et al., 2014) have been reported to elicit amygdala activation. In addition, as the amygdala drives dopamine activation in the reward cycle (Pauli et al., 2012), receives afferents from taste areas and somatosensorial and visual stimuli (Rolls, 2016), and projects to the orbitofrontal cortex (Lichtenberg et al., 2017), which is involved in the computation of the value of the stimuli (Gottfried et al., 2003; Rolls, 2016), it is possible that the higher connectivity between these specific regions is associated with a better integration of sensory/taste stimuli and, thus, is related with food intake.

In the same way that the amygdala was implicated in stimuli processing, the left middle occipital and left lingual were implicated in the integration of visual food stimuli (Huerta et al., 2014). Interestingly, in a study where participants were exposed to a single food choice task, the left lingual gyrus and the left calcarine sulcus were more strongly activated in response to high-energy food cues when compared to low-energy choices. Additionally, the middle occipital gyrus activation covaried positively with the proportion of rejected high-energy choices (van der Laan et al., 2014). Altogether, as reduced neural responses to fat, sweet, and umami flavors in the reward circuit, executive control, and gustatory brain regions have been associated with increased intake of highly palatable food (Kure Liu et al., 2019), it is likely that the combined contribution of food-related stimuli integration systems is associated with food intake. Additional experiments are required to elucidate the relationship between the MedDiet, structural connectivity, and eating behaviors. If these findings are confirmed for future studies, these results might help to develop more effective interventions.

Note that the lateralization to the right reported in the white matter integrity is in line with that of Pelletier et al. (2015), where FA differences were represented on the right hemisphere (Pelletier et al., 2015). In terms of the structural connectivity, the network found on the left hemisphere might be related to the handedness of the studied population. That is, the functional lateralization of the taste processing was found to be lateralized according to the participants’ handedness, i.e., right-handed participants displayed a stronger activation of the left insula (Faurion et al., 1999). As the participants of this study are all right-handed, that can partially explain our findings. Alternately, a lateralization was also hypothesized in terms of dietary decision making. More specifically, when individuals were asked to make decisions regarding which foods they would like to consume, increased activity in the left dorsolateral prefrontal cortex was shown to be associated with healthier decisions (Lowe et al., 2019).

The strengths of the present study include the use of a dietary assessment tool whose scoring allows comparisons between studies and a multimodal MRI approach. The scoring of MEDAS is based on cutoff points that better represented the observed dose–response relationships between the MedDiet and a significant protection against myocardial infarction in a case-control study (Martinez-Gonzalez et al., 2002). This is in opposition to tools that use population sex-specific median, i.e., the scoring of each item of the questionnaire depends on the sample’s median consumption of each item. This scoring method makes it difficult to compare results between studies. Additionally, the multimodal MRI approach offers valuable understanding of the relationship between brain structure and dietary habits. Nonetheless, the results presented here should be interpreted with caution for several reasons. First, self-report of food intake can incur in underreporting of unhealthy food items and the opposite for healthy items (Zuniga and McAuley, 2015). However, the exclusion of participants with implausible dietary intake did not change the results (Supplementary Tables S10–14). At the same time, MEDAS does not capture certain eating habits such as more processed foods, ready meals, and savory snacks, which might yield different scores (Papadaki et al., 2018); yet, this does not seem to be the case as ascertained by the 24-h dietary recalls. The researchers were not blinded to the group assignment, i.e., low MEDAS vs. high MEDAS, during the statistical analysis. However, the groups were formed based on a cutoff previously reported to indicate high adherence to the MedDiet. Also, dietary assessment was measured on an average of 18 months (range 16–25 months, 10.2% > 19 months) prior to the MRI scan and neuropsychological assessment. Yet, the consistency of the dietary pattern was confirmed by a non-significant paired *t*-test between the two moments. Second, the small sample size (*n* = 76) limits the generalization of the findings, precluding the use of sensitivity analyses to exclude participants with lower cognitive scores. Yet, it should be noted that none of the participants at baseline had a diagnosis of dementia nor mild cognitive impairment. Third, ApoE e4 status was not ascertained; however, its reported prevalence in the Portuguese population is lower (Rodrigues et al., 2005) than for other cohorts (Singh et al., 2006). As for the probabilistic tractography connectivity analyses, this approach does not determine an absolute fiber count; instead, this method informs about the connection strength or whether the number of axons exceeds a certain value (Jones et al., 2013), and the results are deemed valid when compared to direct white matter neuron tracing (Khalsa et al., 2014). Last, despite adjustment for several factors, residual confounding cannot be ruled out as in all observational studies.

In summary, we have shown that high adherence to the MedDiet is associated with higher executive functioning scores and preserved white matter integrity of white matter bundles linking limbic regions with the prefrontal cortex and taste-processing regions. Our results, together with findings from research on food decision making and weight loss programs, suggest that the low adherence to the MedDiet might be associated with brain alterations of regions involved in the processing and integration of taste, reward, and decision making, which could interfere with normal food–reward–decision function.

## Data Availability Statement

The datasets generated for this study are available on request to the corresponding authors.

## Ethics Statement

The studies involving human participants were reviewed and approved by Comissão de Ética para a Saúde da Administração Regional de Saúde do Norte, Comissão de Ética do Hospital de Braga, Comissão Nacional de Proteção de Dados. The patients/participants provided their written informed consent to participate in this study.

## Author Contributions

NS and NCS conceived the study. TC, NS, and CP-N recruited the participants. TC and LA performed the psychological assessments. CP-N collected the dietary data. PM, JS, and RM acquired the MRIs. PSM computed the cognitive scores. BR, RM, and AC preprocessed the MRI data. BR and AC analyzed the data. BR wrote the first draft of the manuscript. All authors contributed to the following and final versions of the manuscript.

## Conflict of Interest

The authors declare that the research was conducted in the absence of any commercial or financial relationships that could be construed as a potential conflict of interest.

## Footnotes

1 www.switchbox-online.eu/

2 http://surfer.nmr.mgh.harvard.edu

3 https://fsl.fmrib.ox.ac.uk/fsl/fslwiki/FDT

4 https://fsl.fmrib.ox.ac.uk/fsl/

5 https://sites.google.com/site/bctnet/comparison/nbs

6 http://www.nitrc.org/projects/bnv/
