# Supplementary material for: Higher Adherence to the Mediterranean Diet Is Associated With Preserved White Matter Integrity and Altered Structural Connectivity
Source: Front Neurosci. 2020 Aug 12;14:786. doi: 10.3389/fnins.2020.00786 (PMC7434945; doi:10.3389/fnins.2020.00786)
Supplement: Supplementary file 1 [file Data_Sheet_1.pdf]

Supplementary Material

Supplementary Table S1. Association between adherence to the Mediterranean diet, as measured by MEDAS as continuous variable, and executive functioning scores

				95% Confidence interval	
	B	β	p-value	Lower bound	Upper bound
**(Constant)**	.462		.75	-.2.450	3.373
**Gender[a]**	-.104	-.051	.63	-.537	.328
**Age, years**	-.019	-.148	.23	-.0251	-.012
**Education, school years**	.111	.448	<.0001	.057	.165
**GDS, score**	-.033	-.214	.05	-.066	.0004
**Occupation status[b]**					
**Retired**	-.224	-.098	.46	-.826	.378
**Unemployed**	-.093	-.026	.81	-.806	.661
**Total Energy, Z score**	-.035	-.035	.74	-.246	.176
**BMI, kg/m²**	-.007	-.023	.81	-.063	.049
**MEDAS score**	.118	.232	.02	.020	.215

[a] – Female coded as 0 (reference),
[b] – Reference category “Employed”.
Test statistic - $F(9, 62) = 7.25$, $p < .0001$, adj. $R^2 = .45$.
Abbreviations: GDS – geriatric depression scale; BMI – body mass index; MET – metabolic equivalent; MEDAS – Mediterranean Diet Assessment Screener.

Supplementary Table S2. Association between adherence to the Mediterranean diet, as measured by MEDAS as continuous variable, and memory scores.

				95% Confidence interval	
	B	β	p-value	Lower bound	Upper bound
**(Constant)**	1.35		.48	-2.424	5.126
**Gender[a]**	-.413	-.182	.15	-.974	.148
**Age, years**	-.044	-.299	.04	-.085	-.003
**Education, school years**	.082	.035	.02	.012	.152
**GDS, score**	-.050	-.292	.023	-.094	-.007
**Occupation status[b]**					
**Retired**	.273	.107	.49	-.061	.085
**Unemployed**	.273	.088	.47	-.507	1.053
**Total Energy, Z scores**	-.013	-.011	.93	-.286	.261
**BMI, kg/m²**	.012	.037	.75	-.061	.085
**MEDAS, score**	.121	.213	.06	-.006	.247

[a] – Female coded as 0 (reference).
[b] – Reference category Employed.
Test statistic: $F(9, 62) = 3.67$, $p = .001$, adj. $R^2 = .27$.
Abbreviations: GDS – geriatric depression scale; BMI – body mass index; MET – metabolic equivalent; MEDAS – Mediterranean Diet Assessment Screener.

Supplementary Table S3. Association between adherence to the Mediterranean diet, as measured by MEDAS as dichotomous variables, i.e. High MEDAS Vs Low MEDAS, to total grey matter volume (model 1).

				95% Confidence interval	
	B	β	p-value	Lower bound	Upper bound
**(Constant)**	.075		.36	-.086	.235
**ICV, L**	.295	.602	<.0001	.211	.380
**Time between assessments, months**	.006	.154	.078	-.001	.012
**MEDAS[a]**	.026	.175	.051	-.0001	.058

[a] – MEDAS coded as 0 for low adherence and 1 for high adherence.
Results are presented as B (unstandardized coefficient) and β (standardized coefficients).
Abbreviations: ICV – intracranial volume; MEDAS – Mediterranean Diet Assessment Screener.

Supplementary Table S4. Association between adherence to the Mediterranean diet, as measured by MEDAS as dichotomous variables, i.e. High MEDAS Vs Low MEDAS, to total grey matter volume (model 2).

				95% Confidence interval	
	B	β	p-value	Lower bound	Upper bound
**(Constant)**	.320		.002	.122	.519
**ICV, L**	.271	.551	<.0001	.192	.349
**Time between assessments, months**	.004	.099	.226	-.002	.010
**Age, years**	-.003	-.299	.0004	-.004	-.001
**MEDAS[a]**	.025	.168	.043	.001	.050

[a] – MEDAS coded as 0 for low adherence and 1 for high adherence.
Results are presented as B (unstandardized coefficient) and β (standardized coefficients).
Abbreviations: ICV – intracranial volume; MEDAS – Mediterranean Diet Assessment Screener.

Supplementary Table S5. Association between adherence to the Mediterranean diet, as measured by MEDAS as dichotomous variables, i.e. High MEDAS Vs Low MEDAS, to total grey matter volume (model 3).

				95% Confidence interval	
	B	β	p-value	Lower bound	Upper bound
**(Constant)**	.460		<.0001	.261	.658
**ICV, L**	.229	.466	<.0001	.126	.332
**Age, years**	-.002	-.245	.001	-.003	-.008
**Time between assessments, months**	.003	.076	.301	-.003	.008
**Gender[a]**	.024	.178	.102	-.005	.053
**Education, school years**	.002	.120	.123	-.001	.005
**BMI, kg/m^2**	-.004	-.214	.009	-.007	-.001
**MEDAS[b]**	.010	.067	.39	-.013	.034

[a] – Female coded as 0 (reference).
[b] – MEDAS coded as 0 for low adherence and 1 for high adherence.
Results are presented as B (unstandardized coefficient) and β (standardized coefficients).
Abbreviations: ICV – intracranial volume; MEDAS – Mediterranean Diet Assessment Screener.

Supplementary Table S6. Test statistics for the multiple regression analysis to test the association between adherence to the Mediterranean diet and the total brain volumes.

	TGMV (L)	TWMV (L)	HypoV (mm^3)
**Model 1**	R^2=.49, F (3,72)=23.5,p<.001	R^2=.58, F (3,72)=32.7,p<.001	R^2=.05, F (3,72)=1.21,p=.31
**Model 2**	R^2=.58, F (4,71)=24.2,p<.001	R^2=.69, F (4,71)=39.58,p<.001	R^2=.34, F (4,71)=9.13,p<.001
**Model 3**	R^2=.68, F (7,68)=20.4,p<.001	R^2=.70, F (7,68)=23.0,p<.001	R^2=.40, F (7,68)=6.61,p<.001

Supplementary Table S7. Regions of significant fractional anisotropy, High adherence to the MedDiet > Low adherence to the MedDiet.

		Peak Coordinates			Mean MEDAS<10	Mean MEDAS≥10	Cohen's D
**Cluster index**	**Cluster Size**	**X**	**Y**	**Z**			
**1**	12699	35	-31	4	0.472	0.513	1.6612

MedDiet = Mediterranean diet, MEDAS = Mediterranean Diet Assessment Screener. The x, y, and z coordinates correspond to the MNI space.
1 - Body, splenium and genu of corpus callosum, right cerebral peduncle, right anterior limb of internal capsule, right posterior limb of internal capsule, right retrolenticular part of internal capsule, right anterior corona radiata, right superior corona radiata, right posterior corona radiata, right posterior thalamic radiation (include optic radiation), right sagittal stratum (include inferior longitudinal fasciculus and inferior fronto-occipital fasciculus), right external capsule, right fornix (cres)/stria terminalis, right Superior longitudinal fasciculus, right uncinate fasciculus.

Supplementary Table S8. Regions of significant radial diffusivity, Low adherence to the MedDiet > High adherence to the MedDiet.

		Peak Coordinates			Mean MEDAS<10	Mean MEDAS≥10	Cohen's D
**Cluster index**	**Cluster Size**	**X**	**Y**	**Z**			
**1**	17623	34	-31	6	0.4854	.05200	1.516

MedDiet = Mediterranean diet, MEDAS = Mediterranean Diet Assessment Screener. The x, y, and z coordinates correspond to the MNI space.
1 - Middle cerebellar peduncle, pontine crossing tract, genu of corpus callosum, body and splenium of corpus callosum, left and right corticospinal tract, , left and right superior cerebellar peduncle, left and right cerebral peduncle, right anterior limb of internal capsule, left and right posterior limb of internal capsule, , left and right retrolenticular part of internal capsule, right anterior corona radiata, right and left superior corona radiata, left and right posterior corona radiata, right and left posterior thalamic radiation (include optic radiation), , left and right sagittal stratum (include inferior longitudinal fasciculus and inferior fronto-occipital fasciculus), left and right external capsule, right cingulum (cingulate gyrus), left and right fornix, right superior longitudinal fasciculus, left and right uncinate fasciculus and right tapetum.

Supplementary Table S9. Regions of significant mean diffusivity, Low adherence to the MedDiet > High adherence to the MedDiet.

		Peak Coordinates			Mean MEDAS<10	Mean MEDAS≥10	Cohen's D
**Cluster index**	**Cluster Size**	**X**	**y**	**z**			
**1**	18189	35	-31	4	0.4978	0.5271	1.313

MedDiet = Mediterranean diet, MEDAS = Mediterranean Diet Assessment Screener. The x, y, and z coordinates correspond to the MNI space.
1 – Middle cerebellar peduncle, pontine crossing tract, body of corpus callosum, splenium of corpus callosum, left and right corticospinal tract, left medial lemniscus, left and right superior cerebellar peduncle, left and right cerebral peduncle,right anterior limb of internal capsule, left and right posterior limb of internal capsule, ledt and right retrolenticular part of internal capsule, right anterior corona radiata, right and left superior corona radiata, left and right posterior corona radiata, ledt and right posterior thalamic radiation, left and right sagittal stratum (include inferior longitudinal fasciculus and inferior fronto-occipital fasciculus), left and right external capsule, left and right cingulum (cingulate gyrus), left and right fornix (cres) / Stria terminalis, left and right superior longitudinal fasciculus, right uncinate fasciculus and right tapetum.

Analyses – excluding the participants with implausible intake

Supplementary Table S10. Demographic and cognitive profile of the full cohort and grouped by low vs high adherence to the Mediterranean diet excluding participants with implausible intake.

	**All**	**Low MEDAS**	**High MEDAS**	**Test statistic**
**Sample size**	74	54	20	
**Age, years**	66.7 (7.62)	67.0 (7.60)	65.7 (7.79)	F_{welch}(1,33.3) =0.46, p=.50
**Female, n, (%)**	37 (50.0)	30 (55.6)	7 (35.0)	$X^2_{1,74}$=2.47, p=.19
**Education, school years**	5.81 (4.10)	5.2 (3.63)	7.6 (4.85)	F_{welch}(1,27.3)=4.00, p=.06
**Energy intake, kcal**	2132(824)	2040 (704)	2377(1061)	F_{welch}(1,25.6)=1.73, p=.20
**GDS, score**	9.56 (6.79)	10.3 (6.93)	7.63 (6.19)	F_{welch}(1,39.1)=2.26, p=.14
**BMI, kg/m²**	29.4 (3.66)	30.1(3.53)	27.6 (3.46)	F_{welch}(1,35.6)=2.37, p=.01
**Physical activity, METmin/week**	561 (584)	496 (549)	735 (653)	F_{welch}(1,29.5)=2.13, p=.16
**Smoking habits, n, (%)**				
**Non-smoker**	50 (67.6)	39 (72.2)	11 (55.0)	$X^2_{1,74}$=2.47, p=.27
**Former smoker**	20 (27.0)	12 (22.2)	8 (40.0)	
**Smoker**	4 (5.4)	3 (5.6)	1 (5.0)	
**Occupation status, n, (%)**				
**Employed**	12 (16.2)	8 (14.8)	4 (20.0)	$X^2_{1,74}$=.58, p=.82
**Retired**	56 (75.7)	41 (75.9)	15 (75.0)	
**Unemployed**	6 (8.1)	5 (9.3)	1 (5.0)	
**Neuropsychological assessment**				
**Memory scores**	-0.189 (1.12)	-0.382 (1.02)	0.330 (1.24)	F_{welchv}(1,29.1)=5.28, p=.03
**Executive functioning**	0.076 (1.02)	-0.093 (0.944)	0.531 (1.09)	F_{welch}(1,30.2)=5.13, p=.03

Values are mean (SD) unless otherwise specified. Abbreviations: GDS – geriatric depression scale; BMI – body mass index; MET – metabolic equivalent.

Supplementary Table S11. Association between adherence to the Mediterranean diet, as measured by MEDAS as continuous variable, and executive functioning scores excluding the participants with implausible intake.

				**95% Confidence interval**	
	B	β	p-value	Lower bound	Upper bound
**(Constant)**	.594		.40	-.2.450	3.373
**Gender[a]**	-.085	-.042	.71	-.537	.367
**Age, years**	-.019	-.141	.24	-.051	.013
**Education, school years**	.108	.441	<.0001	.053	.163
**GDS, score**	-.035	-.229	.045	-.069	-.001
**Occupation status[b]**					
**Retired**	-.241	-.102	.45	-.869	.387
**Unemployed**	-.106	-.029	.79	-.889	.678
**Total Energy, Z scores**	-.048	-.037	.74	-.332	.236
**BMI, kg/m^2**	-.009	-.032	.75	-.066	.048
**MEDAS**	.111	.218	.03	.011	.211

[a] – Female coded as 0 (reference),
[b] – Reference category “Employed”.
Test statistic - $F(9, 69) = 7.05$, $p < .0001$, adj. $R^2 = .44$
Abbreviations: GDS – geriatric depression scale; BMI – body mass index; MET – metabolic equivalent; MEDAS – Mediterranean Diet Assessment Screener.

Supplementary Table S12. Association between adherence to the Mediterranean diet, as measured by MEDAS as continuous variable, and memory scores excluding the participants with implausible intake.

				95% Confidence interval	
	B	β	p-value	Lower bound	Upper bound
**(Constant)**	1.619		.40	-.2.195	5.432
**Gender[a]**	-.389	-1.336	.19	-.970	-.002
**Age, years**	-.043	-.290	.04	-.084	-.012
**Education, school years**	.076	.276	.04	.005	.147
**GDS, score**	-.054	-.316	.02	-.098	.010
**Occupation status[b]**					
**Retired**	.226	.086	.58	-.582	1.034
**Unemployed**	.317	.079	.53	-.691	1.325
**Total Energy, Z score**	-.017	-.011	.93	-.382	.349
**BMI, kg/m^2**	.008	.024	.83	-.066	.081
**MEDAS**	.110	.192	.09	-.019	.238

[a] – Female coded as 0 (reference),
[b] – Reference category "Employed".
Test statistic - $F(9, 60) = 3.65$, $p = .001$, adj. $R^2 = .26$
Abbreviations: GDS – geriatric depression scale; BMI – body mass index; MET – metabolic equivalent; MEDAS – Mediterranean Diet Assessment Screener.

Supplementary Table S13. Association between the Mediterranean diet scores and global brain volumes excluding the participants with implausible intake.

	TGMV (L)		TWMV (L)		HypoV (mm^3)	
	B	p	B	p	B	p
**Model 1**	.025	.071	-.015	.216	-1556	.135
**Model 2**	.025	**.048**	-.015	.134	-1538	..080
**Model 3**	.009	.447	-.022	.053	-1015	.268

MEDAS coded as 0 for low adherence and 1 for high adherence.
Results are presented as B (unstandardized coefficient) and p-values. Test statistics for each model in Table S13.
Model 1 – adjusted for ICV, Model 2 – adjusted for ICV and age, Model 3 – adjusted for ICV, age, years of education, gender and BMI. Abbreviations: TGMV – total grey matter volume; TWMV – total white matter volume; HypoV – hypointensities volumes.

Supplementary Table S14. Test statistics for the multiple regression analysis to test the association between adherence to the Mediterranean diet and the total brain volumes excluding the individuals with implausible intake.

	**TGMV (l)**	**TWMV (l)**	**HypoV (mm^3)**
**Model 1**	R^2=.49, F (3,70)=21.9,p<.001	R^2=.58, F (3,70)=32.2,p<.001	R^2=.05, F (3,70)=1.2,p=.32
**Model 2**	R^2=.57, F (4,69)=22.5,p<.001	R^2=.68, F (4,69)=51.6,p<.001	R^2=.34, F (4,69)=8.7,p<.001
**Model 3**	R^2=.68, F (7,66)=19.5,p<.001	R^2=.70, F (7,66)=22.3,p<.001	R^2=.40, F (7,66)=6.3,p<.001
